# A Deep Learning Method for 3D Object Classification and Retrieval Using the Global Point Signature Plus and Deep Wide Residual Network

**DOI:** 10.3390/s21082644

**Published:** 2021-04-09

**Authors:** Long Hoang, Suk-Hwan Lee, Ki-Ryong Kwon

**Affiliations:** 1 Department of Artificial Intelligence Convergence, Pukyong National University, Busan 48513, Korea; hoanglongdtvt2001@gmail.com; 2 Department of Computer Engineering, Dong-A University, Busan 49315, Korea; skylee@dau.ac.kr; 3 Department of IT Convergence and Application Engineering, Pukyong National University, Busan 48513, Korea

**Keywords:** Global Point Signature Plus, Deep Wide Residual Network, 3D object classification and retrieval, multimedia contents processing and retrieval

## Abstract

A vital and challenging task in computer vision is 3D Object Classification and Retrieval, with many practical applications such as an intelligent robot, autonomous driving, multimedia contents processing and retrieval, and augmented/mixed reality. Various deep learning methods were introduced for solving classification and retrieval problems of 3D objects. Almost all view-based methods use many views to handle spatial loss, although they perform the best among current techniques such as View-based, Voxelization, and Point Cloud methods. Many views make network structure more complicated due to the parallel Convolutional Neural Network (CNN). We propose a novel method that combines a Global Point Signature Plus with a Deep Wide Residual Network, namely GPSP-DWRN, in this paper. Global Point Signature Plus (GPSPlus) is a novel descriptor because it can capture more shape information of the 3D object for a single view. First, an original 3D model was converted into a colored one by applying GPSPlus. Then, a 32 × 32 × 3 matrix stored the obtained 2D projection of this color 3D model. This matrix was the input data of a Deep Residual Network, which used a single CNN structure. We evaluated the GPSP-DWRN for a retrieval task using the Shapnetcore55 dataset, while using two well-known datasets—ModelNet10 and ModelNet40 for a classification task. Based on our experimental results, our framework performed better than the state-of-the-art methods.

## 1. Introduction

The 3D models, including rigid models (CAD models) and non-rigid models (scanned human data), cover extra information than the 2D models, with various applications in a self-driving car, facial recognition, and human–computer interaction. 3D classification and retrieval tasks are foundational research topics in computer vision and graphics, so they have crucial 3D applications, e.g., virtual reality, medical diagnosis, and digital preservation. For example, in the digital preservation area, the arbitrary 3D objects could be captured from the given colorful models, using the techniques in the 3D retrieval task [1]. Two challenges in the 3D model analysis are the choice of network structure and the usage of 3D data representation. It is compulsory to use 3D classification and retrieval methods for effectively managing large-scale 3D objects, due to the rapid growth in the number of 3D objects.

Deep learning techniques rapidly developed in various tasks in image processing, such as gene identification [2,3], biomedical/medical imaging [4,5], and so on [6], in recent years. For instance, many studies propose various deep networks such as Multi-View Convolutional Neural Networks (MVCNN) [7], 3D ShapeNets [8], and PointNet [9] for 3D model classification and retrieval, by using deep learning neural networks and the available 3D large-scale data. View-based methods such as MVCNN have a superior performance among current methods (View-based, Voxelization, and Point Cloud methods). These view-based methods combine a trainable system with 2D projection attributes adopted by the Convolutional Neural Networks (CNN), so they achieved sophisticated results for 3D model recognition at that time. The satisfactory performance of MVCNN inspired various researchers to develop an integrated deep learning model, which had benefits from the projected view-images for 3D object classification and retrieval tasks. However, these approaches created the 2D projection directly from the 3D object, so the number of views increased to compensate for the information loss. The number of views depended on the feature concatenation, the virtual camera setting, and the complex parallel CNN structure. Zeng et al. [10] present the virtual camera setting, in which the first camera setup in Reference [7] allowed to obtain the multiple projected-images for inputs of the MVCNN model. The input 3D model of the first camera setup was required to be placed vertically to a constant axis, which is the requirement of most 3D model datasets, including ModelNet40. Twelve virtual cameras were placed around each 3D model to capture 12 views because each camera focused at its center, with a $30^{o}$ angle to the horizontal axis. Qier An et al. [11] mathematically demonstrated the optimal camera configuration in 3D reconstruction.

**Figure 1:**
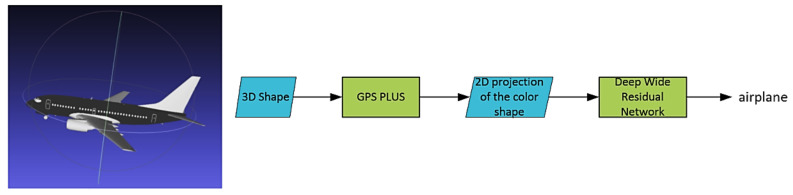
The GPSP-DWRN.

One issue with the MVCNN is that there is not enough spatial information from a few views, especially for a single view. To handle this issue, we propose a novel descriptor—Global Point Signature Plus (GPSPlus), which captured both local and global features of the 3D shape, then created the 2D projection of the 3D object after applying GPSPlus. GPSPlus increased more spatial information for every single 2D projection, classified by a Deep Wide Residual Network (DWRN). Figure 1 shows the structures of Global Point Signature Plus-Deep Wide Residual Network (GPSP-DWRN).

The core idea of GPSPlus was inspired by the geometric feature extraction method and complementary techniques that handle spatial loss in 3D reconstruction. Reference [12] proposes an algorithm on the geometric feature extraction of the new point cloud. The algorithm could fit the point cloud boundary with high precision, even with local spatial losses on the boundary. Another study (see reference [13]) presented both techniques—Terrestrial Laser Scanning (TLS) and photogrammetry, which completed each other for generating a complete model of the heritage complexes. Photographs mainly extract texture/radiometric information, while laser systems produce more accurate geometric information. Additionally, laser systems cannot easily reach areas where photogrammetry can get more information.

GPSPlus uses the complement techniques, combining local and global geometric feature extraction. Local or global geometry alone cannot sufficiently characterize the entire model in almost all cases. Global features specify the full shape, while local features encode the point’s local geometry. The complement of both local geometry and global geometry makes the GPSPlus encode more geometric information (more spatial information) of 3D objects.

In this article, our contributions are as follows:

- We introduce a novel descriptor GPSPlus for extracting both local and global information of the 3D object. We use a single view instead of multi-views in the existing view-based methods, because GPSP-DWRN provides more spatial information for the 3D model representation on a single view. The GPSP-DWRN could work with the low hardware computer resource, due to the minimum input size of the 2D projection image.
- A Deep Wide Residual Network learns the extracted color feature from the 3D object effectively, after applying GPSPlus. Our derivation of GPSP-DWRN is novel, to the best knowledge of the authors.
- Based on our experimental results on challenging datasets, the GPSP-DWRN is superior and more efficient than other well-known approaches.

The paper structure is as follows. We review related works in Section 2, then describe the method in Section 3. Section 4 presents the relevant experimental settings, experimental results, and discussion. Finally, Section 5 draws concluding remarks.

## 2. Related Work

This section reviews some related works. There are three categories—Point Cloud methods, Voxelization methods, and view-based methods, based on the diversity of data structures in 3D model-representing techniques.

- Point Cloud Methods: A raw unranked point cloud represents the 3D data. These methods usually analyze the neighborhood from every point with a given radius, to extract features [14]. For example, PointNet++ [15] applies transformations that are order-invariant to every point, to generate a vector of features for the cloud. The local attributes generated are sampled, grouped, and used for scene classification and segmentation. Point-Voxel CNN (PVCNN) [16] combines the representation of sparse data with voxelized convolutions to improve data access performance. Point-Voxel Convolution (PVConv) accumulates neighbor points with convolutions based on voxel, converts points into voxel grids, and transforms them back. The authors included point-based transformations to obtain more detailed features. VoteNet [17], a Hough voting based-on method, uses PointNet++ layers as the backbone and chooses the points with the corresponding attributes as the seed to generate clusters of vote-based object instances. These clusters, with their categories, are finally transferred into 3D bounding boxes.
- Voxelization: The original point cloud discretizes into input data. Points are measured in the neighborhood distance for grouping into various clusters. Every voxel is expressed commonly as 0 for the presence or 1 for the absence in points in the space represented. The authors of ModelNet introduced 3D ShapeNets, used a cubic voxel to present the data, and generated the features by applying 3D convolutions. Similarly, VoxNet [18] was classified by applying a 3D CNN to the volumetric representation. References [19,20] reduced memory consumption and improved performance by combining octree descriptions and 3D convolution performance. Another approach, PointGrid [21], used a point-quantization technique to create grid cells with a constant number of points. This technique saves the coordinates of points for the representation improvement of local geometric objects.
- View-based methods: View-based methods are now popular in recent years, due to the independence of 3D reconstruction and possible application with multi-view representation [22]. Ansary et al. [23] selected representative views using X-means and then measured the similarity between pairwise 3D objects by applying Bayesian models. Shih et al. [24] introduced a feature descriptor that is shape-invariant under transformation for retrieval. Murase et al. [25] changed pose and illumination automatically for using multiple views to represent 3D models. Wang et al. [26] solved the retrieval problem by using the group sparse coding. The query object was constructed again by the view sets of each applicant shape, then the restoration error was considered to be the similarity measurement for retrieval. Reference [27] used representative views to construct the weighted bipartite graph to measure similarity. Liu et al. [28] suggested extracting an attribute view set based on a graph and matching approach for 3D retrieval. Another work, MVCNN, was proposed by Su et al., which first applied 2D ConvNets on 2D views of 3D objects for extracting visual features from separate 2D projections. The view pooling layer conducted a full stride channel-wise max pooling to obtain a unified feature vector. Lastly, the fully-connected layer predicted the class of one 3D object. Gao et al. [29] introduced a hyper-graph outline strategy for the retrieval task. Reference [30] proposed a matching method based on a clique-graph with the multi-model to learn the architectural features of the 3D model. Bai et al. [31] presented a search engine of 3D objects in real time, which depended on multiple-views using a twice-inverted file to improve the multi-view matching method, and learn the local distribution of 3D objects. Kanezaki et al. [32] introduced Rotationet, taking a 3D object’s multi-view images as inputs, and simultaneously estimated the class and the pose of this object.

## 3. Methodology

The color 3D shape, generated from the original 3D one by applying the GPSPlus, was used to create the 2D projection (see Figure 1). This 2D projection is the input of the Deep Wide Residual Network for feature extraction and classification. Next, we fully describe how the heat kernel and the original global point signature (GPS) were combined to create the GPSPlus and preserve their good properties in Section 3.1. Then, the Deep Wide Residual Network is described in Section 3.2.

### 3.1. Gpsplus

First, we reviewed the property of the heat kernel. While the local connectivity or topology of the graph determined the heat kernel for a short time, the solution to the heat equation for a long time measured the global geometry of the manifold. The recent discretization technique of the Laplacian Eigenspectrum made computational methods efficient and robust. The surface was modeled as a homogeneous vibrating membrane from spectral theory for shape analysis (see References [33,34]), and therefore Equation (2) in Reference [35] described its harmonic behavior. The powerful Spectral methods used for solving differential equations played a crucial role in object representation, due to the pose-invariant-property of the Laplace–Beltrami operator. The Eigenspectrum, in contrast, could generate a shape representation that provided a quantitative method for calculating surface differences and then analyzing the shape. As is well-known in the literature, this shape representation is called GPS embedding. The GPS’s coordinates specify attributes on a manifold, characterize geometric features of an object or similarities of two distinct manifolds. These GPS coordinates in Reference [33] are:

(1) $$GPS\left(v\right)=\left(\frac{{Ø}_{1}\left(v\right)}{\sqrt{\lambda_{1}}},\frac{{Ø}_{2}\left(v\right)}{\sqrt{\lambda_{2}}},\ldots ,\frac{{Ø}_{i}\left(v\right)}{\sqrt{\lambda_{i}}},\ldots ,\frac{{Ø}_{S}\left(v\right)}{\sqrt{\lambda_{S}}}\right),$$

where ${Ø}_{i}\left(v\right),\;i=\bar{1,S}$ is the value of the eigenfunction ${Ø}_{i}$ at point v; $\lambda_{i}$ is the eigenvalue; and S is the numbers of eigenvalues. The GPS is invariant under isometry, unique to the signature for every point on the manifold. We introduce a novel shape descriptor, namely GPSPlus, a combination of the heat kernel and the original GPS. We choose t = 1 in the heat kernel equation to eliminate the global information and keep the local one, because the GPS already captured the global one. As a result, GPSPlus combines the locality of the heat kernel and the globality of GPS. The GPSPlus preserves all properties of the GPS and the heat kernel due to the linear convex combination. The coordinates of GPSPlus are defined as

(2) $$\left(\left(\alpha e^{-\lambda_{1}}+\frac{\beta }{\sqrt{\lambda_{1}}}\right){Ø}_{1}\left(v\right),\left(\alpha e^{-\lambda_{2}}+\frac{\beta }{\sqrt{\lambda_{2}}}\right){Ø}_{2}\left(v\right),\ldots ,\left(\alpha e^{-\lambda_{S}}+\frac{\beta }{\sqrt{\lambda_{S}}}\right){Ø}_{S}\left(v\right)\right),$$

where α=β=0.5. First, a Laplacian matrix from a 3D mesh is derived, then its eigenvalues and the corresponding eigenvectors are calculated before applying Equation (2) to the 3D mesh (see Reference [36]). Given a vertex v on the mesh, we define its GPSPlus as

(3) $$GPSPlus\left(v\right)=\overset{S}{\underset{i=1}{\sum }}\left(\alpha e^{-\lambda_{i}}+\frac{\beta }{\sqrt{\lambda_{i}}}\right){Ø}_{i}\left(v\right).$$

We choose the number of eigenvalues S=3, which leads to the robust result in practice. The shape information is voided because the first eigenvector is constant with all vertices of the 3D mesh [37]. We ignore the first eigenvalue and choose three adjacent eigenvalues to calculate the corresponding eigenfunctions and GPSPlus values, respectively. GPSPlus is surprisingly concise because only a few eigenvalues are used to obtain an accurate shape description.

**Figure 2:**
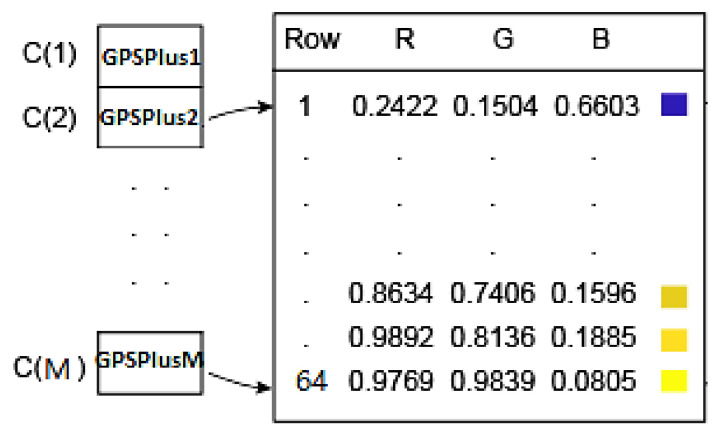
The colormap vs. M vertices.

Then, the GPSPlus vector of a 3D mesh is determined by

(4) $$\left[\begin{array}{c} \begin{array}{c} GPSPlus_{1}\\ GPSPlus_{2} \end{array}\\ \vdots \\ GPSPlus_{M} \end{array}\right],$$

where each $GPSPlus_{j},j=\bar{1,M}$ is obtained from applying the GPSPlus in Equation (3) to each vertex $V_{j}$. The GPSPlus feature vector forms a colormap. Every value of GPSPlus, controls each value of vertex and color (see Figure 2).

Suppose that the smallest and the largest values, namely GPSPlus2 and GPSPlusM, are at the second and the $M^{th}$ vertices. Then, $GPSPlus_{2}$ and $GPSPlus_{M}$ are transformed to the first and the last rows of the colormap, respectively, as shown in Figure 2.

**Figure 3:**
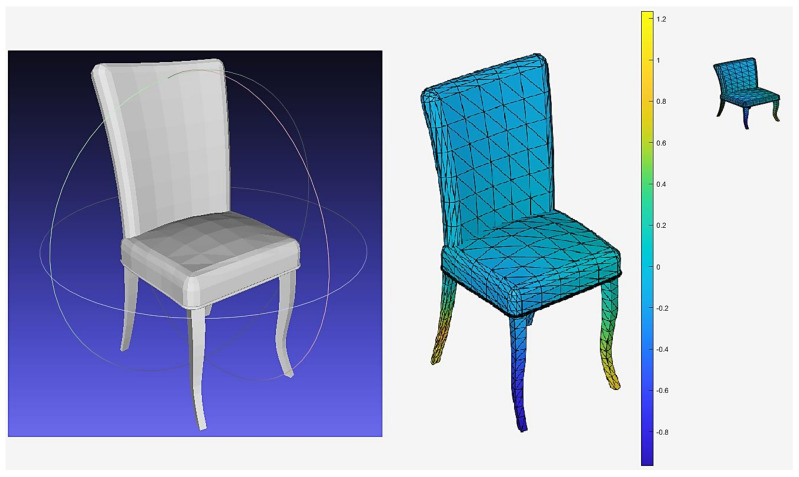
(**Left**)—the original 3D chair. (**Right**)—the 2D view 32 by 32 by 3. (**Middle**)—the 3D color model using GPSPlus value.

Figure 3 shows the color model converted from the original model after applying GPSPlus to the 3D model. A 2D view of the 3D color model is captured and stored by using a 32 × 32 × 3 matrix.

### 3.2. Deep Wide Residual Network

Firstly, the DWRN develops from the idea of skip connection. Now, we discuss the skip connection. The stacking of convolution layers, which usually construct CNN, allows a given network to learn from lower-level features in a hierarchical setting. However, a given layer is assumed to only connect with its two adjacent layers.

The information from earlier layers might be lost during backpropagation [38], so this assumption is shown to be less optimal. Reference [38] proposes skip connections, which allow for deeper networks while maintaining a low number of parameters and preserving the feature information across all layers. The input of a given layer might be a sum of previous layers.

**Figure 4:**
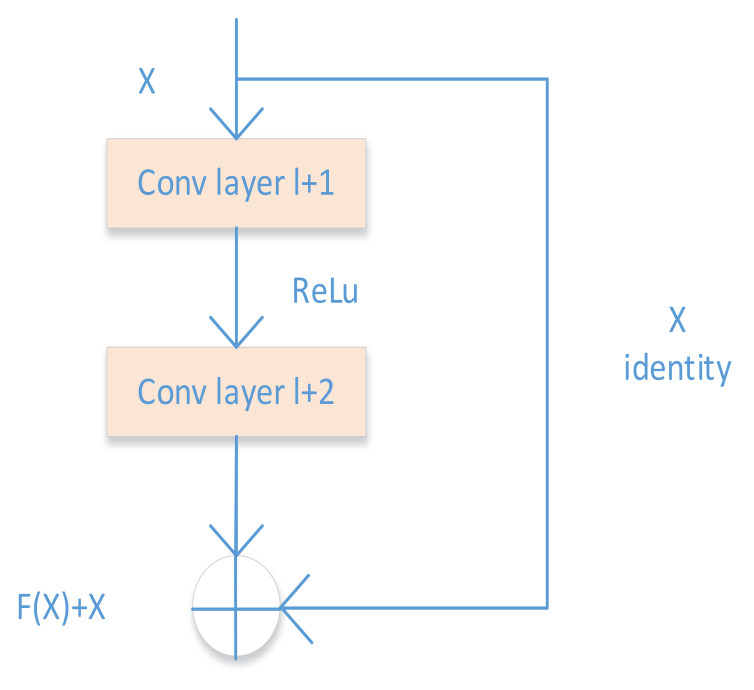
Skip connections with r = 2.

Figure 4 shows an example of skip connections with r = 2, which forms the basic unit of the residual network. This basic unit, referred to as a residual module [39], is combined to create the entire network. The skip connection copies and adds the input of layer l to the output of layer (l + r). This approach makes the gradient flow efficient because it uses a super-highway and the skip connections in the gradient backpropagation algorithm.

**Figure 5:**
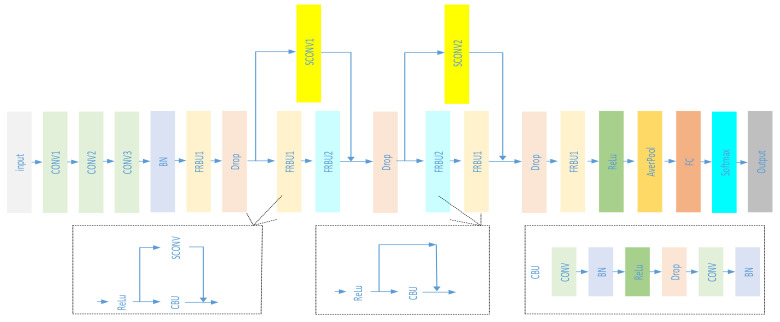
The proposed Deep Wide Residual Network.

Secondly, the GPSP-DWRN uses the basic module convolutional block unit (called CBU in Figure 5), which was stacked to build the networks. The convolutional block unit consists of the convolutional layer (Conv), the batch normalization (BN) [40], the rectified linear unit (ReLU) [41], the drop out layer (Drop) [42], Conv, and BN. We used the BN and the Drop layer for the following reasons.

Batch normalization first reduced overfitting through regularization, accelerated training by a magnitude order, and predicted more stability from network output. Activations, normalized by Batch normalization across a mini-batch, subtract their mean, divided by their standard deviation. Some possibly higher activations might cause a network to be less stable and the subsequent layers are abnormal, therefore, normalization is crucial. Ioffe et al. [40] ensured that the network always produces activations with the desired distribution for any parameter values. Therefore, the Batch Normalization Layer is inserted before ReLu or any other activations, but right after a Conv Layer.

Next, we added a dropout layer after the activation layer. Dropout was first introduced in Reference [42], then adopted by many successful architectures (see References [43,44]). A Dropout model is the weighted output average of prediction or estimation from different models. Dropout is mostly applied on top layers with many parameters to prevent overfitting and feature coadapting. The hidden layer nodes, which possibly occur randomly, could be ignored from selecting randomly in the dropout. Hence, such a unique training network defines a new model. Any two hidden-nodes do not repeatedly overlap in models, so updating the weights without relying on the interplay of fixed nodes bypasses potential interactions among features. Ignoring these hidden layer nodes can reduce the computational cost and overfitting induced by joining nodes, without restriction from these ignored nodes (see Reference [45]).

In the GPSP-DWRN, the extra convolution layer on the skip connection ensured the number of filters on the left side and the right side of the residual block was equal, allowing to add input to the residual block without any errors. Extra BN is added right after the skip Conv, so-called the SCONV module, as shown in Figure 5.

Figure 5 shows that our network has two more modules, the so-called residual block unit (FRBU1 and FRBU2). Typically, there is one skip for one residual module. The novelty is that the GPSP-DWRN stacks FRBU1 and FRBU2 together, adding the SCONV module to extend the width of a residual network module for its performance improvement.

The kernel size in the GPSP-DWRN was 1 × 1 for convolution layers in the SCONV module and 3 × 3 for all other convolution layers. Table 1 shows the number of stride and the filters are equal between two convolution layers in the same CBU.

## 4. Experiment

### 4.1. Datasets

The GPSP-DWRN is evaluated on datasets—ModelNet10, ModelNet40 [8], and ShapeNetCore55 [46]. The ModelNet dataset includes 127,915 CAD objects from 662 object classes, and we evaluated on it two subsets—ModelNet10 and ModelNet40. The full shapenet dataset has 3135 classes with more than 3,000,000 CAD objects, and we evaluated its subset—ShapeNetCore55.

ModelNet10: ModelNet10 has 4866 objects from 10 categories, in which the training, testing use 3991 and 908 models, respectively. We use the same split for training and testing, as mentioned in Reference [8] for the fair comparison.

ModelNet40: ModelNet40 contains 12,311 CAD objects from 40 classes, including 10 of ModelNet10. The whole datasets are split into two parts—one with 9841 training models and one with 2468 testing models (see Reference [8]).

ShapeNetCore55: ShapeNetCore55, providing 51,300 3D shapes from 55 classes, were split 70%, 10%, 20% for the training/validation/test in our experiments (see Reference [46]).

### 4.2. Evaluation

For retrieval, every shape in the test dataset was chosen as a query for our experiments. Popular measures, such as NN, FT, ST, Precision, Recall, F-Measure, ANMRR, DCG, and mAP, were evaluated for 3D model retrieval. Next, these criteria were fully described.

Precision and Recall: An item in the retrieved lists is positive or negative if it is in the same or different category with the target model. We calculate the precision and the recall for each entry in the lists. The precision at an entry is the percentage of positive items of this entry. The recall at an entry is the ratio of positive items of this entry to the minimum value between the maximally allowed retrieved list length and the total numbers of objects in the category. The Precision and Recall can fully evaluate the retrieval performance.

(5) $$\mathrm{Precision}=\frac{\mathrm{True}\;\mathrm{Positives}}{\mathrm{True}\;\mathrm{Positives}+\mathrm{False}\;\mathrm{Positives}},\;$$

(6) $$\mathrm{Recall}=\frac{\mathrm{True}\;\mathrm{Positives}}{\mathrm{True}\;\mathrm{Positives}+\mathrm{False}\;\mathrm{Negatives}},\;$$

Nearest Neighbor (NN): NN indicates the performance of the nearest neighbor classifier. NN is the proportion of the closest matches that are in the same category as the query. High scores mean good retrieval performance.

First Tier (FT): For a class with N objects, FT is defined as the recall for the maximal (N − 1) matches in the ranking list, K = recall (N − 1). FT indicates the lowest K that the recall possibly includes 100% of the objects in the query. Higher values indicate better matches, with a score of 100% for an ideal matching result.

Second Tier (ST): Similarly, ST is measured depending on the size of the query’s category, K = recall (2 × (N − 1)).

The F measure (F): The F-score is calculated based on the precision and recall at each entry.

(7) $$F=\frac{2*P_{20}*R_{20}}{P_{20}+R_{20}},\;$$

where $P_{20}$ and $R_{20}$ denote the output of the precision and recall of the top 20 retrieval, respectively.

Discounted Cumulative Gain (DCG): A statistic indicates that correct retrievals ahead of the ranking list are more important than the corresponding results at the back of the list. Suppose that a user prefers to count objects near the fronting list. NDCG is specified by relevant grades when comparing category and subcategory in the query and the retrieval. The scores are as follows: 0 for no match, 1 for a correct match for the category, but incorrect for the subcategory, 2 for both category and subcategory that belong to a category, 3 for a perfect match between category and subcategory in the query, and the retrieval. The subcategory is used only in NDCG. The DCG and the NDCG are used for ModelNet, and ShapeNet, respectively.

Average Normalized Modified Retrieval Rank (ANMRR): ANMRR is a measure based on the ranking facts of related objects amongst the retrieved ones. A higher value of ANMRR indicates worse retrieval.

Mean Average Precision (mAP): mAP is a broad measure for solving the single-point value problem of Precision, Recall, and F-measure.

There are two metrics—macro-averaged and micro-averaged metrics for evaluating ShapeNetCore55, as shown on the official website. The first metric calculates an unweighted average over the whole dataset using the aforementioned metrics, whereas the second one computes a weighted mean regarding the objects’ numbers from different classes.

### 4.3. Implementation Details

In ModelNet and Shapnet, objects are re-meshed or not if the face number is more or less than 3600, due to the dissimilarity in the number of vertices of the model in the identical category. For instance, the airplane category had a maximum of 2,583,632 faces and a minimum of 1253 faces. The re-meshed model and the original one were similar because we still kept the shapes and excluded insignificant information. The role of the re-meshing process was the same as the technology in the image or audio compressing. A user is less likely to distinguish between the MP3 compressed sound and the original wave sound. Maintaining the formation of the opening dataset and re-meshing for some objects helped to create a new dataset. All experimentations were implemented on the PC i7 8700, 16GB memory, 1070 GPU (8GB memory), MATLAB (9.9 (R2020b), Natick, MA, USA). We used the initial learning rate at 0.1 and divide by half after every 60 epochs, the momentum at 0.9 for network training, and the mini-batch at 32 for SGD. The hyperparameters values were chosen because the numerical results were optimal, based on our various experiments.

### 4.4. The Comparison on ModelNet Dataset

We recently collected publicly available results of the classification and retrieval on two datasets—modelnet and shapenet—from other methods, for a comparison with GPSP-DWRN. Different evaluation criteria are favored for various 3D shape datasets and competitions, so we still use their convention for evaluation. We use NN, FT, ST, F-measure, DCG, ANMRR, mAP, and ACC on ModelNet 40 dataset and Modelnet 10 dataset while using P@N, R@N, F1@N, NDCG, and mAP on the ShapeNetCore55 dataset.

Table 2 shows that GPSPlus and Deep Wide Residual Network (GPSP-DWRN) outperformed all other methods in terms of classification accuracy on the ModelNet10 dataset. The GPSP-DWRN outperformed most methods except SCFN and MVHFN in the classification task on the ModelNet40 dataset. The difference in the number of views and inputs of the network might lead to the underperformance of the GPSP-DWRN, as compared to the SCFN and MVHFN. First, both SCFN and MVHFN used eight-views, compared with one view in the GPSP-DWRN. Secondly, GPSP-DWRN worked with the color image size 32 by 32 by 3, while both SCFN and MVHFN were in line with the black and white one of size 224 by 224 and of size 224 by 224, respectively. Class unit 8 utilized one byte per element of memory in Matlab, so it took 0.383 MB of memory to store eight images of size 224 by 224 for one 3D shape and 4713 MB of memory for a whole ModelNet40 dataset. Additionally, the GPSP-DWRN used a matrix of size 32 × 32 × 3 to describe each 3D object. As a result, it consumed only 36 MB of memory for the ModelNet40 dataset, which was one percent of the memory size used by the SCFN and MVHFN methods. On the contrary, the GIFT method used an image size of 224 by 224 with 64 views, hence, it cost 37702 MB of memory for the ModelNet40 dataset, which was 1000 times the memory of the GPSP-DWRN. As a result, GIFT required the highest hardware resources with an Intel (R) Xeon (R) CPU (3.50 GHz), 64GB RAM, and 4 GTX NVIDIA TITAN X. Finally, SCFN, MVHFN, and GIFT caused the complexity of the deep learning network structure, due to the usage of parallel CNN structures with eight-branch CNN and sixty-four-branch CNN.

The benefits of the GPSP-DWRN were that GPSP-DWRN employed a single CNN instead of a multi-branch CNN and used the GPSPlus to capture a single 2D view of a 3D object, improving accuracy without using many views. The local information of the GPSPlus helped to increase the ability of deep learning-based methods in 3D object recognition. Deep learning-based methods improved their performance thanks to the capacity of extraction and usage of local information. The combination of the single CNN network and Deep Wide Residual Network in the GPSPlus allowed learning both global features and local features of objects.

**Figure 6:**
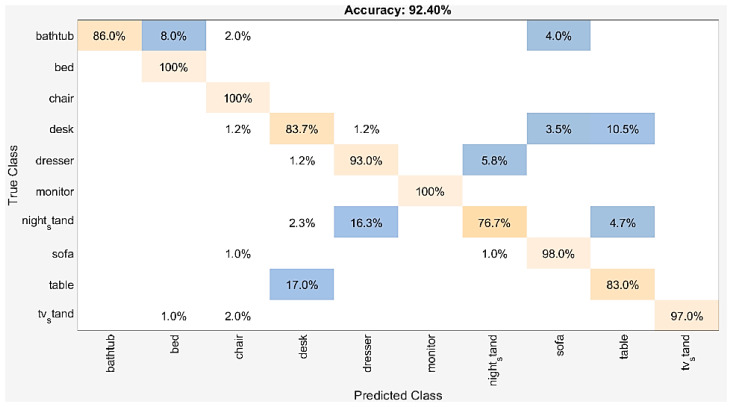
The confusion matrix for dataset: ModelNet10.

**Figure 7:**
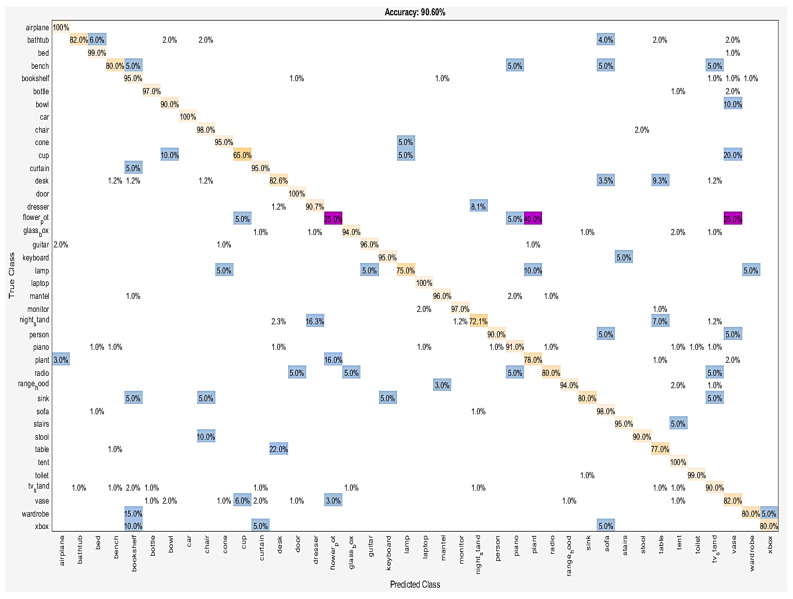
The confusion matrix for dataset: ModelNet40.

Statistical results of each category for the classification task are reported in detail. Figure 6 and Figure 7 show the percentage of correctly classified objects over whole objects, in each class. Nightstand and Table categories were classified worst with 76.7% and 83.0% accuracy, respectively, on ModelNet10. Two nightstand shapes were misclassified as the desk, fourteen other as the dresser, and four others as the table. Seventeen table shapes were misclassified as the desk. The visual similarities between these categories were the main reason for these failure cases. On ModelNet40, six classes with an accuracy lower than 80% were the cup (65%), the flowerpot (25%), the lamp (75.0%), the nightstand (72.1%), the plant (78%), and the table (77%).

Among the top three confusions, 25% of flowerpot was misclassified as a vase, 40% flowerpot as the plant, and 22% table as the desk. Some of these pairs are hard to distinguish by the method, even by humans, due to their similarities.

The activations of the fully connected layer of the DWRN for each input 3D model were used as the corresponding descriptor for that object to perform the retrieval task. These activations were in line with the softmax function for classification or retrieval by similarity measurement of pairwise 3D models. The Euclidean distance measured similarity in our experiments.

**Figure 8:**
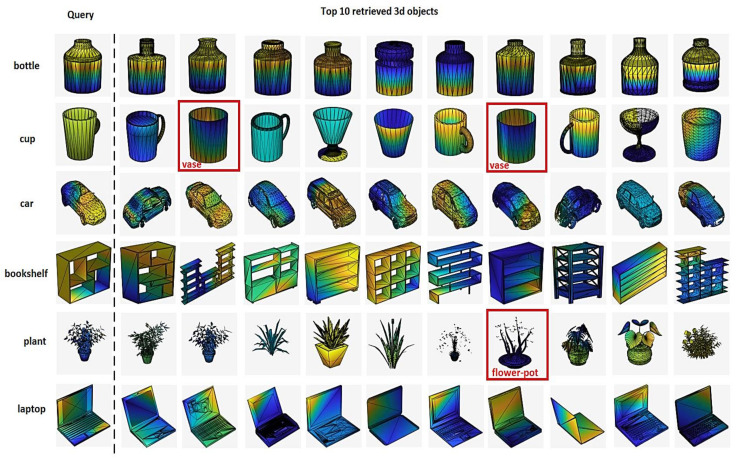
The top 10 retrieved objects for the ModelNet40 dataset.

As shown in Figure 8, the query objects are on the first left column, including six classes—the bottle, the cup, the car, the bookshelf, the plant, and the laptop, while the top 10 retrieved ones are on the right, depending on the distances to the query objects in the embedded space. All objects shown by a red box were retrieved wrong. The overall retrieval results were acceptable. Based on the query objects, the GPSP-DWRN produced correct results in most categories. All top results were accurate, except bottles, cars, bookshelves, and laptops. Additionally, there was some irrelevant results for cups and plants. We argued that the similarities between these 3D objects were due to errors. For example, it was hard to distinguish pairs—the cup and the vase; the plant and the flowerpot manually, due to their similarities.

Table 3 and Table 4 compare retrieval performance between the GPSP-DWRN and other methods, based on seven metrics—NN, FT, ST, F-measure, ANMRR, DCG, and mAP.

The proposed GPSP-DWRN performed the best among the methods on five metrics, even on the most three crucial metrics—DCG, ANMRR, and mAP. The mAP is the most representative index to analyze the retrieval performance. Table 4 shows that the GPSP-DWRN method increased the retrieval performance of the mAP measure by 15.7%, as compared to the MVCNN method on the ModelNet40 dataset.

### 4.5. The Comparison on Shapenet Datasets

The Shape Retrieval Contest (SHREC) is an annual famous shape retrieval contest. In this subsection, our experiments were implemented on the large-scale shape retrieval benchmark ShapeNetCore55 from SHREC 2017 and it consistently proved the accuracy and the efficiency of our method. We used the training set for classification and the validation set for the retrieval tasks in our experiments. Table 5 shows all deep-learning-based methods, except the Li_ZFDR. As shown in Table 5, GPSP-DWRN is more accurate than other state-of-the-art algorithms in terms of the five indicators, including micro P@N, micro mAP, micro NDCG, macro mAP, and macro NDCG, but was relatively bad in the other five measures on ShapeNetCore55.

Performance measures—the precision and the recall showed opposite indicators. Therefore, the F1 score was the harmonic average of the precision and the recall, possibly leading to the limitation of a single value problem. Thus, the sampling distribution would easily affect the F1 score. The mAP was the regional area under the precision-recall curve; the mAP could solve this problem. The mAP was considered the most crucial criterion to measure retrieval performance. As a result, the GPSP-DWRN obtained the lower R@N, F1@N but higher P@N and mAP. P@N and R@N were the precision and recall of the top 20 retrievals, respectively. F1@N was calculated from Equation (7). Another important evaluation criterion, NDCG, considered the location of the retrieval output in its list.

Almost all multi-view based-methods in Table 5, including Zhou_Improved_GIFT, Kanezaki_RotationNet, Thermos_MVFusionNet, Tatsuma_ReVGG, Deng_CMVGG5-6DB, SHREC16-Bai_GIFT, SHREC16-Su_MVCNN, SCFN, and MVHFN, used the latest advanced technology in 2D image recognition to obtain outperformance in 3D object retrieval. The GPSP-DWRN, a combination of Global Point Signature Plus and Deep Wide Residual Network, showed a significant improvement in the retrieval results by around 10.5% in micro mAP, compared to MVCNN. GPSP-DWRN showed a higher accuracy than other existing methods—NDCG (0.7–61.6%), macro NDCG (2.2–53.2%), micro mAP (1.5–63.8%), and macro mAP (3.4–54.4%), which demonstrate the effectiveness of the GPSP-DWRN on this challenging dataset.

The cup and flowerpot object classes were low accurate in recognition, due to the possibility of the variation of data classes in the study. The higher and lower training samples led to high and low accuracy in object recognition. Oversampling technique increased the number of classes in the training set, reducing the class imbalance and uncertainties in the minority classes. On the contrary, the undersampling technique removed some objects in a higher number of classes. A future research direction is to design those techniques to handle the imbalance between objects.

The object recognition in our study could be applied to a robot with the Microsoft Kinect sensor. The robot ccould extract feature vectors using the proposed method after not recognizing various objects in the scene at the initial step. In the next step, the Kinect sensor captured a 3D object, then the robot labeled the features extracted from this 3D object. Finally, objects could be realized fully by the robot. The GPSP-DWRP would run smoothly with an intelligent robot due to two reasons. First, in terms of memory consumption, GPSP-DWRP consumes less memory at only 1/1000 memory than the GIFT method. Second, in terms of number GPU, GPSP-DWRP uses a single GPU, compared with 4 GPU in the GIFT method. GPSP-DWRP works well with low resource computers and are suitable with an intelligent robot. Although GPSP-DWRP uses less hardware resource, the method still has a higher accuracy when compared to the GIFT method in the modelnet dataset (see Table 2) and performs better on 5/10 metrics in the shapenet dataset (see Table 5).

## 5. Conclusions

3D object classification and retrieval are crucial and challenging tasks in computer vision with various applications in 3D visual processing/Virtual Reality, human–machine interaction, etc. This study proposed a novel method GPSP-DWRN for 3D object classification and retrieval, based on GPSPlus and Deep Wide Residual Network. The GPSP-DWRN can exploit 3D objects better than other view-based methods for both local and global information. Based on our experimental results, GPSP-DWRN showed better performance in the classification and retrieval tasks of 3D objects than some well-known methods on three popular datasets. Our future research will investigate the performance of the GPSP-DWRN in 3D visual processing applications, with the limitation of hardware resources such as robotic-operated 3D understanding with Microsoft Kinect sensor.

## Figures and Tables

**Table 1:** The number of stride and filters in each module.

Module	Unit	Number of Filters	Stride
CONV	CONV1	16	1
	CONV2	32	1
	CONV3	64	1
FRBU1	CBU of First FRBU1	32	1
	CBU of Second FRBU1	64	2
	CBU of Third FRBU1	128	2
	CBU of Fourth FRBU1	128	1
FRBU2	CBU of First FRBU2	64	1
	CBU of Second FRBU2	64	1
SCONV	SCONV1	64	2
	SCONV2	128	2
	SCONV of First FRBU1	32	1
	SCONV of Second FRBU1	64	2
	SCONV of Third FRBU1	128	2
	SCONV of Fourth FRBU1	128	1

**Table 2:** Comparison with different methods on the ModelNet.

Method	Data Format	ModelNet10 ACC mAP		ModelNet40 ACC mAP	
SPH [47]	-	79.8%	-	68.2%	33.3%
LFD [48]	-	79.9%	-	75.5%	40.9%
3D ShapeNets [8]	Voxelization	91.0%	68.2%	75.5%	40.9%
VoxNet [18]	Voxelization	92.0%	-	83%	-
3D-A-Nets [49]	Voxelization	-	-	90.5%	80.1%
PointNet [9]	Point Cloud	-	-	89.2%	-
Effective Point Cloud Encoding Method [41]	Point Cloud	-	-	90.5%	-
Method [50]					
Multi Depth Maps [51]	6 views	-	-	87.8%	-
MVCNN, 12x [7]	12 views	-	-	89.9%	70.1%
MVCNN, metric, 12x [7]	12 views	-	-	89.5%	80.2%
MVCNN, 80x [7]	80 views	-	-	90.1%	70.4%
MVCNN, metric, 80x [7]	80 views	-	-	90.1%	79.5%
SCFN [52]	8 views	92.3%	87.2%	92.2%	83.9%
MVHFN [53]	8 views	-	-	91.6%	80.3%
MVCLN [54]	6 views	92.2%	-	90.6%	-
GIFT [31]	64 views	91.5%	91.1%	89.5%	81.9%
GPSP-DWRN	1 view	92.4%	89.5%	90.6%	85.8%

**Table 3:** Retrieval performance—ModelNet10. Bolding shows the maximum value in each column.

Method	Views	FT	ST	NN	F_Measure	ANMRR	DCG	mAP
SCFN [43]	2	0.769	0.915	0.851	0.303	0.206	0.798	0.816
	4	0.809	0.934	0.888	0.314	0.167	0.838	0.83
	6	0.778	0.904	0.899	0.311	0.199	0.813	0.857
	8	0.832	0.937	**0.911**	**0.327**	0.148	0.855	0.872
GPSP-DWRN	1	**0.862**	**0.966**	0.905	0.309	**0.12**	**0.88**	**0.895**

**Table 4:** Retrieval performance—ModelNet40. Bolding shows the maximum value in each column.

Method	Views	FT	ST	NN	F_Measure	ANMRR	DCG	mAP
SCFN [43]	2	0.635	0.738	0.854	0.302	0.322	0.692	0.775
	4	0.689	0.783	0.887	0.314	0.272	0.743	0.797
	6	0.732	0.829	0.899	0.321	0.233	0.779	0.805
	8	0.814	0.862	**0.903**	**0.332**	0.167	0.829	0.839
MVHFN [44]	2	0.393	0.555	0.651	0.229	0.515	0.457	0.452
	4	0.452	0.627	0.739	0.231	0.374	0.516	0.495
	8	0.769	0.813	0.825	0.321	0.202	0.798	0.803
MVCNN [1]	12	0.671	0.753	0.878	0.311	0.287	0.728	0.701
GPSP-DWRN	1	**0.822**	**0.914**	0.886	0.304	**0.152**	**0.85**	**0.858**

**Table 5:** Retrieval performance—SHAPENETCORE55. Bolding shows the maximum value in each column.

Method	micro					macro				
	P@N	R@N	F1@N	NDCG	mAP	P@N	R@N	F1@N	NDCG	mAP
Kanezaki_RotationNet	81.0%	80.1%	79.8%	86.5%	77.2%	60.2%	63.9%	**59.0%**	65.6%	58.3%
Zhou_Improved_GIFT	78.6%	77.3%	76.7%	82.7%	72.2%	59.2%	65.4%	58.1%	65.7%	57.5%
Furuya_DLAN	81.8%	68.9%	71.2%	66.3%	76.2%	**61.8%**	53.3%	50.5%	47.7%	56.3%
Tatsuma_ReVGG	76.5%	80.3%	77.2%	74.9%	82.8%	51.8%	60.1%	51.9%	49.6%	55.9%
Thermos_MVFusionNet	74.3%	67.7%	69.2%	73.2%	62.2%	52.3%	49.4%	48.4%	50.2%	41.8%
Deng_CM-VGG5-6DB	41.8%	71.7%	47.9%	65.4%	54.0%	12.2%	**66.7%**	16.6%	40.4%	33.9%
DMk_DeepVoxNet	79.3%	21.1%	25.3%	19.2%	27.7%	59.8%	28.3%	25.8%	23.2%	33.7%
Li_ZFDR	53.5%	25.6%	28.2%	19.9%	33.0%	21.9%	40.9%	19.7%	25.5%	37.7%
SHREC16-Su_MVCNN	77.0%	77.0%	76.4%	81.5%	73.5%	57.1%	62.5%	57.5%	64.0%	56.6%
SHREC16-Bai_GIFT	70.6%	69.5%	68.9%	76.5%	64.0%	44.4%	53.1%	45.4%	54.8%	44.7%
SCFN	52.6%	**82.9%**	59.2%	88.2%	80.1%	20.1%	76.4%	21.3%	79.3%	62.5%
MVHFN	75.3%	75.4%	74.7%	88.0%	81.5%	54.0%	57.4%	52.9%	84.7%	74.2%
GPSP-DWRN	**82.6%**	69.1%	71.2%	**89.3%**	**83.0%**	56.7%	36.8%	40.5%	**86.9%**	**77.6%**

## Data Availability

The original ModelNet and Shapenet datasets are available online at https://modelnet.cs.princeton.edu/ and https://shapenet.org/ (accessed on 14 March 2021).
